# Sensitization of Tumors for Attack by Virus-Specific CD8+ T-Cells Through Antibody-Mediated Delivery of Immunogenic T-Cell Epitopes

**DOI:** 10.3389/fimmu.2019.01962

**Published:** 2019-08-21

**Authors:** Julian P. Sefrin, Lars Hillringhaus, Olaf Mundigl, Karin Mann, Doris Ziegler-Landesberger, Heike Seul, Gloria Tabares, Dominic Knoblauch, Andreas Leinenbach, Irene Friligou, Sebastian Dziadek, Rienk Offringa, Valeria Lifke, Alexander Lifke

**Affiliations:** ^1^Discovery Oncology, Roche Innovation Center Penzberg, Roche Pharma Research and Early Development, Penzberg, Germany; ^2^Department of Early Development and Reagent Design, Roche Diagnostics GmbH, Penzberg, Germany; ^3^Large Molecule Research, Roche Innovation Center Penzberg, Roche Pharma Research and Early Development, Penzberg, Germany; ^4^Translational Medicine Oncology, Roche Innovation Center Basel, Roche Pharma Research and Early Development, Basel, Switzerland; ^5^Department of General Surgery, Heidelberg University Hospital, Heidelberg, Germany; ^6^Division of Molecular Oncology of Gastrointestinal Tumors, German Cancer Research Center, Heidelberg, Germany; ^7^Personalized Healthcare Solution, Immunoassay Development and System Integration, Roche Diagnostics GmbH, Penzberg, Germany; ^8^Pharma Biotech Penzberg, Roche Diagnostics GmbH, Penzberg, Germany

**Keywords:** antigen-armed antibodies, antibody-targeted pathogen-derived peptides (ATPP), cancer immunotherapy, immune tolerance, CD8+ T-cell, immunogenicity, targeted therapy

## Abstract

Anti-tumor immunity is limited by a number of factors including the lack of fully activated T-cells, insufficient antigenic stimulation and the immune-suppressive tumor microenvironment. We addressed these hurdles by developing a novel class of immunoconjugates, Antibody-Targeted Pathogen-derived Peptides (ATPPs), which were designed to efficiently deliver viral T-cell epitopes to tumors with the aim of redirecting virus-specific memory T-cells against the tumor. ATPPs were generated through covalent binding of mature MHC class I peptides to antibodies specific for cell surface-expressed tumor antigens that mediate immunoconjugate internalization. By means of a cleavable linker, the peptides are released in the endosomal compartment, from which they are loaded into MHC class I without the need for further processing. Pulsing of tumor cells with ATPPs was found to sensitize these for recognition by virus-specific CD8+ T-cells with much greater efficiency than exogenous loading with free peptides. Systemic injection of ATPPs into tumor-bearing mice enhanced the recruitment of virus-specific T-cells into the tumor and, when combined with immune checkpoint blockade, suppressed tumor growth. Our data thereby demonstrate the potential of ATPPs as a means of kick-starting the immune response against “cold” tumors and increasing the efficacy of checkpoint inhibitors.

## Introduction

In the last decade, cancer immunotherapy has emerged as a highly promising concept to treat cancer with impressive clinical success in recent years. Especially immune checkpoint blockade involving antibody (Ab)-mediated targeting of the CTLA-4 and PD1/PD-L1 pathways has shown unprecedented clinical efficacy through the enhancement of tumoricidal T-cell responses (1). However, only certain tumor types and patients qualify for this therapeutic approach, in that checkpoint inhibition can only work in “hot” tumors that present sufficient numbers of MHC-restricted peptide antigens at their cell surface and that are infiltrated by tumor-reactive T-cells (1).

We have developed a strategy for increasing the immunogenicity of tumors through the delivery of viral peptide epitopes for which the majority of human subjects are known to exhibit memory T-cell responses. Targeted epitope delivery is achieved by conjugating these peptides to Abs that are specific for internalizing, cell surface-expressed tumor antigens. The generation of these antibody-targeted pathogen-derived peptides (ATPPs) involves Ab-peptide conjugation through a cleavable (reducible) disulfide bond, which mediates peptide release in the endosomal compartment upon internalization of the Ab-peptide conjugate. In this manner, we achieved loading of recycling MHC class I molecules in a manner that is independent of the antigen processing machinery (2). Our data demonstrate that ATPPs offer a highly flexible platform for the efficient delivery of a wide variety of T-cell epitopes to tumors, resulting in the sensitization for immune attack by virus-specific T-cells.

## Methods

### Generation of Abs

Sequences of antibodies (Abs) against CD22/Inotuzumab (3), CD79b/Polatuzumab (4), CD138/Indatuximab (5), CDCP1/RG-7287 (6), and PD-1/Nivolumab (7) were derived from available patents. Sequences containing variable regions were generated by gene synthesis with flanking restriction sites (GeneArt) and cloned in mammalian expression vectors comprising the human IgG1 backbone. The P329G LALA mutation was introduced to abolish human IgG1 binding to Fc receptors. Ab chains were transiently co-transfected in HEK-293F cells (Invitrogen) and purified as described (8). Ab homogeneity was analyzed using a Dionex Ultimate 3000 (Thermo Fisher) with a Biosuite TM 250 column (Waters) and by CE-SDS.

### Peptide Synthesis

Peptides were synthesized using standard fluorenylmethoxycarbonyl (Fmoc)-chemistry. The following peptides were generated (species, gene, sequence, HLA class I binding): pEBV_1 (EBV, LMP-2, CLGGLLTMV, HLA-A02:01), pEBV_1_long (EBV, LMP-2, YGPVFMCLGGLLTMV, no HLA class I binding), pEBV_2 (EBV, BMLF-1, GLCTLVAML, HLA-A02:01), pFLU (Influenza A, Nucleoprotein, CTELKLSDY, HLA-A01:01).

### Generation of Ab-Targeted Pathogen-Derived Peptides (ATPPs)

Abs were kept in 0.1 M potassium phosphate buffer containing 150 mM NaCl, pH 7.5. Eight equivalents of N-Succinimidyl 3-(2-pyridyldithio)-propionate (SPDP, Pierce) were added. After 2 h reaction time, the derivatized Ab was dialyzed against 0.1 M potassium phosphate buffer containing 150 mM NaCl and 10 mM EDTA at pH 7.0. 6 equivalents of the respective peptide were added to the derivatized Ab, reacted overnight and dialyzed against storage buffer (20 mM Histidine, 150 mM NaCl). Conjugates containing a non-cleavable thioether linker were generated according to the protocol above using succinimidyl iodoacetate (SIA, Pierce) instead of SPDP.

### Synthesis of Anti-CDCP1-FRET Conjugate

Generation of the fluorescence resonance energy transfer (FRET) conjugate was adapted from Yang et al. (9). A peptide used for linking the different components of the FRET conjugate with the sequence Acetyl-Cys-Lys-Ala-Glu-βAla-Glu-βAla-Glu-Azidohomoalanine was prepared using standard Fmoc-chemistry on a TentaGel R RAM resin. The peptide was cleaved from the resin and purified by preparative HPLC. A solution of sulforhodamine B acid chloride was reacted with 1 equivalent of cysteamine dithiopyridyl in dimethylformamide containing 4 equivalents of trimethylamine. 2 equivalents of the resulting rhodamine dithiopyridine were incubated with Cys-containing peptide in phosphate buffer at pH 7.5. After 90 min 2 equivalents of BODIPY FL NHS ester were added and after additional incubation for 90 min the final product (SS-FRET peptide) was purified by preparative HPLC.

In order to generate the αCDCP1-FRET conjugate, αCDCP1 Ab was incubated with 5 equivalents of cyclooctyne NHS ester (SX-A1028, Synaffix) in phosphate buffer at pH 8.3 and purified by gel filtration. The resulting αCDCP1-cycloctyne conjugate was incubated with 30 equivalents of the SS-FRET peptide in phosphate buffer at pH 7.0 for 3 h, in order to generate the αCDCP1-FRET conjugate. The product was finally purified by gel filtration.

### Mass Spectrometric Analysis of ATPP Peptide Labeling Rates

ATPPs were subjected to mass spectrometric analysis, in order to determine peptide labeling rates. ATPPs and unlabeled reference Ab were deglycosylated with N-glycosidase F and subsequently measured. Analytics were performed using an LC-ESI-MS (Waters) HPLC system and a reverse-phase column with a water-acetonitrile gradient and ESI-TOF-MS measurement and detection. MS data analysis was performed using MassLynx Software (Waters).

### Cell Lines and Culture

Cancer cell lines were purchased from the American Type Culture Collection (ATCC). Cell lines were verified as pathogen-free and identity was confirmed by means of single nucleotide polymorphism PCR or short tandem repeat analysis. MDA-MB231 cells were cultured in RPMI1640 + 10% FBS + 2 mM L-Glutamine, HCT-116 in McCoy's 5A + 10% FBS + 2 mM L-Glutamine, A375 in DMEM + 10% FBS + 2 mM L-Glutamine, PC-3, BxPC-3 and U266B1 in RPMI1640 + 10% FBS + 2 mM L-Glutamine + 1 mM Sodium Pyruvate + 10 mM HEPES. All media and supplements were ordered from Life technologies. Cell lines were passaged for a maximum of 5 times.

### PBMC Preparation and CD8+ T-Cell Enrichment

Peripheral blood mononuclear cells (PBMCs) were isolated by Ficoll gradient centrifugation from EDTA-blood of healthy donors. CD8+ T-cell enrichment was achieved by means of RosetteSep™ Human CD8+ T-cell Enrichment Cocktail (StemCell) according to the manufacturer's protocol. Enrichment quality was checked by post-sort flow cytometric analysis.

### Preparation of Peptide-Specific CD8+ T-Cell Cultures

PBMCs were cultured in RPMI1640 medium containing 8% human serum, 1 mM Sodium Pyruvate, 1 mM non-essential amino acids, 2 mM L-Glutamine, 50 μM β-mercaptoethanol and 1 μM of the respective peptide. After 3 days, fresh peptide and 20 ng/mL IL-15 and 500 ng/mL soluble, Fc-fused IL15-Rα (R&D Systems) was added. Cells were restimulated every 2 weeks with autologous, irradiated (40Gy), peptide-pulsed PBMCs. Cells were expanded every 4–5 days with fresh medium supplemented with IL-15 and receptor, as indicated above. Peptide specific expansion of T-cells was monitored by flow cytometric analysis using MHC-peptide pentamers on a regular basis. Cultures used for functional assays were >70% CD8+, >60% peptide-specific and 10–14 days after last restimulation.

### Flow Cytometry

Non-specific binding of Abs was suppressed by incubation with human TruStain FcX™ Fc receptor blocking solution (BioLegend). For analysis of isolated tumor xenografts, cells were additionally incubated with TruStain fcX™ (anti-mouse CD16/32 Ab, BioLegend). 1 μg/mL DAPI was added to stain dead cells. Cells were labeled with the following Abs (clone) or MHC-peptide pentamers (HLA molecule, peptide sequence): CD3 (SK7), CD4 (OKT4), CD8α (HIT8a), CD45 (HI30), CD138 (DL-101), CD279/PD1 (EH12.2H7), CD318/CDCP1 (CUB1), HLA-A2 (BB7.2), HLA-A1/36 (8.L.104), HLA-A1/11/26 (8.L.101), pEBV_1 pentamer (HLA-A02:01, CLGGLLTMV), pEBV_2 pentamer (HLA-A02:01, GLCTLVAML), pFLU pentamer (HLA-A01:01, CTELKLSDY). Almost all Abs (including isotype controls) were purchased from BioLegend, except for the HLA-A1 Abs, which were obtained from Abcam. Pentamers were provided by ProImmune. Cells were analyzed using the BD Biosciences Canto II. Ab quality was validated in pilot experiments and gating was performed using isotype controls. The FlowJo (Treestar) software was used to analyze flow cytometry data and to calculate mean fluorescence intensity (MFI).

### Internalization Assay

Cells were harvested by means of Accutase and incubated with 10 μg/mL αCDCP1 Ab, αCDCP1-EBV_1 or the respective human IgG Isotype (Sigma) for 30 min on ice. Cells were washed and incubated in medium at 4 or 37°C. After *t* = 0, 0.5, 1, 2, 4 and 24 h, cells were stained with secondary Ab for 30 min on ice (polyclonal goat anti-human IgG, Life technologies) to detect non-internalized ATPPs at the cell surface. 1 μg/mL DAPI was added to discriminate dead cells. Flow cytometry was performed using the BD Biosciences Canto II and data was analyzed by means of the FlowJo (Treestar) software. Percent internalization for each time-point was calculated as follows: (MFI at 37°C / MFI at 4°C) × 100.

### T-Cell Activation and Cytotoxicity Assays

1.5 × 10^4^ target cells were incubated for 24 h with ATPPs and/or control substances in tumor cell medium. Cells were washed and peptide-specific effector T-cells or PBMCs were added in AIM-V CTS medium (Gibco) at an effector-to-target ratio of 3:1 or 20:1, respectively, if not specified otherwise. In case of MHC-blocking experiments, αHLA-ABC Ab (clone W6/32, BioLegend) was added 10 min prior to T-cells.

For real-time analysis of target cell killing the xCELLigence analyzer (Roche) was used. Target cell killing in % was calculated as [(cell index of target cells—cell index treatment)/(cell index of target cells] × 100.

After 24 h supernatants were collected and used to assess T-cell activation by Interferon-γ (IFNγ) enzyme-linked immunosorbent assay (ELISA) and target cell death by lactate dehydrogenase (LDH) measurement. T-cell activation was investigated by quantifying IFNγ released into the supernatant by human IFNγ DuoSet ELISA system (R&D Systems).

The Cytotoxicity Detection Kit (Roche) was used according to the manufacturer's instructions in order to measure LDH activity. Absorbance was detected at 492 nm (reference: 620 nm) using a Tecan infinite 200Pro Reader. Maximum LDH release was determined by lysing target cells with 1% Triton X-100 (Sigma-Aldrich). Percentage of lysis was calculated as [(LDH release during treatment – LDH release of target cells) / (maximum LDH release – LDH release of target cells) × 100].

For time-lapse imaging of tumor cell killing, tumor cells were labeled with 2 μM CMFDA (Life technologies) and time-lapse fluorescence imaging was performed in a 37°C, 5%CO_2_, 95% humidity chamber on a Leica SP8 microscope using hybrid detectors. Imaging conditions were as follows: 63 × /1.20 water immersion lens with sequential acquisition for each channel using white light laser excitation at 488 nm and emission at 492–553 nm for CMFDA or excitation at 561 nm and emission at 567–670 nm for PKH-26.

### FRET Analysis by Confocal Microscopy

1 × 10^5^ MDA-MB231 cells were pulsed with 10 μg/mL of αCDCP1-FRET conjugate for 30 min on ice. Cells were washed twice with PBS and incubated for *t* = 0, 2, or 18 h in cell culture media at 37°C, 5%CO_2_ and subsequently fixed with 4% PFA. To investigate donor (BODIPY) and Ab co-localization Alexa Fluor 647 conjugated αIgG (H+L) Ab (Life technologies) was used. Confocal microscopy was performed on a Leica SP8 microscope using hybrid detectors. Imaging conditions were as follows: 100x/1.46 N.A. oil immersion lens with sequential acquisition for each channel using white light laser excitation at 488 nm and emission at 492–553 nm for BODIPY or 561 nm and 567–670 nm for Rhodamine. Alexa Fluor 647 was excited at 647 nm and detected at 653–700 nm. Endosomal images were subjected to deconvolution using Huygens Essential (Scientific Volume Imaging B.V.).

### Mouse Tumor Xenograft *in vivo* Study

Four to 6 week old female CIEA NOG mice were obtained from Taconic Biosciences (Denmark). All mice were housed in the pharmacology department at the Roche Innovation Center Munich (Penzberg, Germany) in compliance with national and international regulations. 5 × 10^6^ MDA-MB231 cells were s.c. injected into the right flank per mouse. When tumors reached ~70 mm^3^, mice were assigned to treatment and control groups by randomized allocation. Tumor volume was regularly monitored by means of blinded caliper measurement. αCDCP1-EBV_1 (20 mg/kg/week) and αPD1 Ab (5 mg/kg/week) were administered intraperitoneally every third day, starting on day 21. On day 22, 5 × 10^6^ pEBV_1-specific CD8+ T-cells, *in vitro* expanded from human PBMCs, were injected intravenously into the tail vein together with 1.5 μg IL-15 and 7 μg sIL15Ra-Fc (R&D Systems).

For post-mortem flow cytometric analyses, tumors were harvested and cut into small pieces by means of a scalpel and digested for 15 min at 37°C in RPMI1640 Medium containing 1 mg/mL Dispase II (Roche), 1 mg/mL Collagenase IV (Sigma Aldrich) and 0.1 mg/mL DNase I (Roche). The digest was strained through a 70 μm nylon mash, washed, and subsequently subjected to staining for flow cytometry.

### Statistics

Statistical analysis of experimental data was performed by means of the Prism (GraphPad) software using one-way ANOVA followed by Tukey's multiple comparison test. Results were considered statistically significant if *p* < 0.05.

## Results

### Internalization and Endosomal Delivery of T-Cell Epitopes by ATPPs

**Figure 1:**
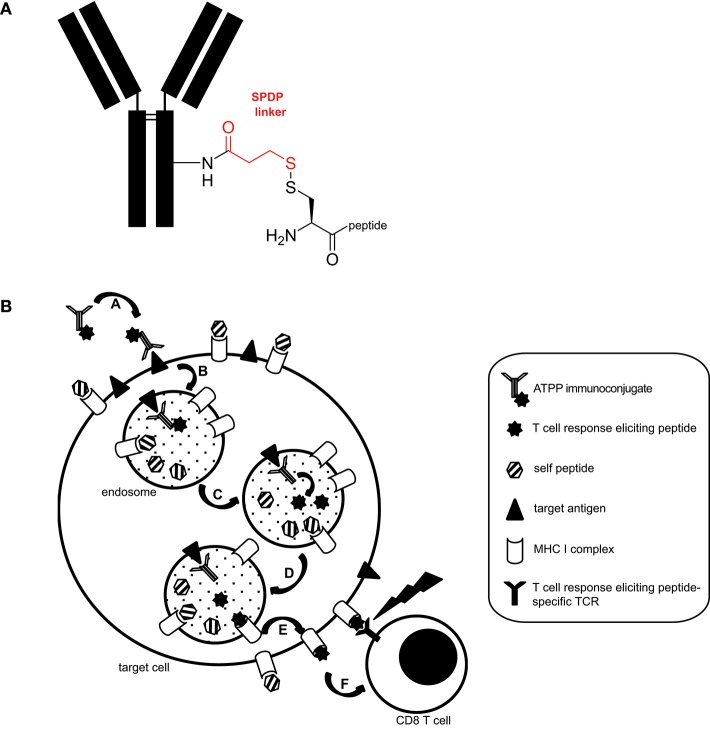
Structure and proposed mode of action of ATPP immunoconjugates. **(A)** Representative structure of ATPP immunoconjugates. Free lysine residues on the antibody serve to attach an SPDP linker (red) via an amide bond. The immunogenic peptide is connected to the linker via a cysteine using a disulfide bond. The cysteine can reside in the middle, on the C- or N-terminus of the peptide. **(B)** Proposed model for the mode of action of ATPPs. A: Binding of ATPP to cell surface expressed target antigen. B: Internalization of ATPP into endosomal compartment. C: Release of T-cell response eliciting peptide from the immunoconjugate in the endosomal compartment. D: Loading of MHC-I molecules with released peptide. E: Routing of peptide-loaded MHC-I molecules to target cell surface. F: Recognition of peptide-loaded MHC-I molecules on target cell surface by peptide-specific CD8+ cytotoxic T-cells.

We generated a panel of antibody-targeted pathogen-derived peptides (ATPPs) with the aim of selectively delivering immunogenic peptide epitopes to tumor cells. For this proof of concept study, we selected CUB-domain-containing-protein-1 (CDCP1) as the primary antibody (Ab) target, because this integral membrane protein is highly expressed at the cell surface of various cancer types and hence allowed testing of the ATPP approach in multiple tumor cell lines (10–13). Furthermore, our prior work had shown that Ab binding to cell surface expressed CDCP1 leads to rapid internalization of the Ab/antigen complexes (6). Mature, virus-derived MHC class I-restricted peptide epitopes were covalently bound to CDCP1-specific IgG1 antibodies (Abs) with a succinimidyl 3-(2-pyridyldithio)propionate (SPDP) linker (Figure 1A), in order to allow release of the peptide payload in the endosomal compartment. Since disulfide reduction occurs in endosomes (9) and endosomal acidification simultaneously promotes the release of associated (self-) peptides from MHC-I (14), we hypothesized that, upon release, ATPP-derived peptides can bind to recycling MHC-I molecules (15) and be presented on the cell surface (Figure 1B).

ATPPs used in this study were loaded with peptide antigens from EBV and Influenza A, which are known to represent well-defined, naturally processed T-cell epitopes in the context of the highly prevalent HLA-A02:01 and HLA-A01:01 molecules respectively: EBV LMP-2 (pEBV_1), BMLF-1 (pEBV_2), Influenza Nucleoprotein (pFLU). For these epitopes, T-cell memory responses resulting from natural virus encounter can be detected in the vast majority of human subjects positive for these HLA-molecules (16–18). Peptide loading efficiency of the resulting conjugates, as determined by mass spectrometry, ranged between 1.38 and 5.35 for the mature peptide epitopes, depending on the peptide/Ab combination (Supplementary Table 1). To prevent unspecific uptake of ATPPs by — and activation of — Fc-receptor positive antigen presenting cells, the Fc-domain of the αCDCP1 Ab was modified by a P329G-LALA mutation (19).

**Figure 2:**
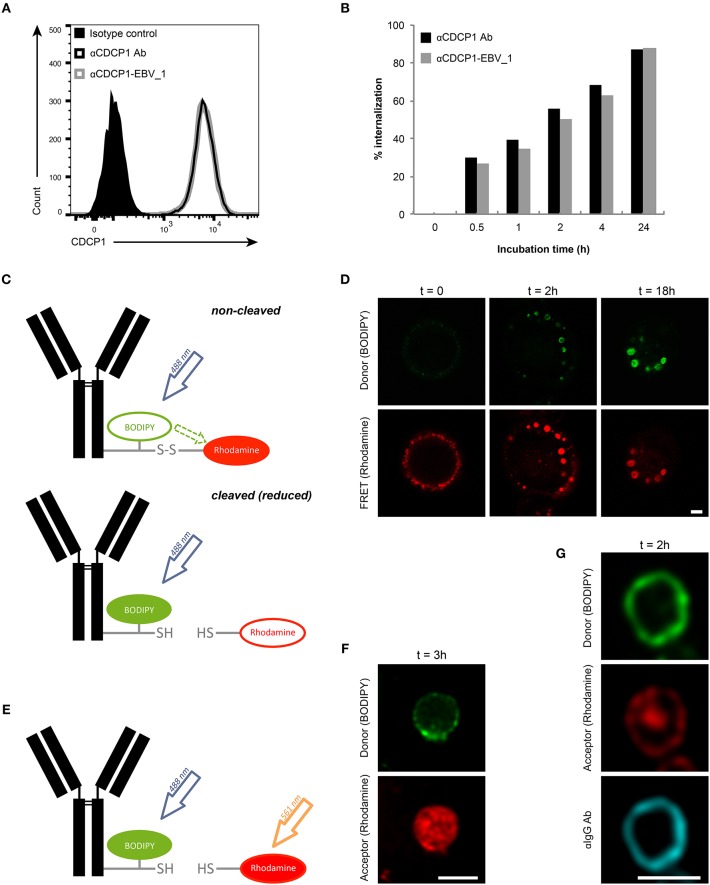
Peptide release from ATPP occurs following internalization in endosomes. **(A)** Binding and **(B)** internalization of αCDCP1 Ab and αCDCP1-EBV_1 ATPP on MDA-MB231 cells as determined by flow cytometry. **(C)** Graphical representation of the αCDCP1 FRET disulfide reporter conjugate. Excitation of the donor chromophore (BODIPY) triggers FRET to the acceptor chromophore (Rhodamine, red signal, upper panel). Release of the acceptor upon disulfide reduction results in green (BODIPY) signals (lower panel). **(D)** Confocal time-lapse imaging of MDA-MB231 cells using the αCDCP1 FRET disulfide reporter conjugate. **(E)** Illustration of separate excitation of donor (BODIPY) and acceptor (Rhodamine) in the cleaved αCDCP1 FRET disulfide reporter conjugate. **(F)** Magnification of an endosome using separate excitation of BODIPY and Rhodamine. **(G)** Magnification of an endosome upon co-staining with αIgG antibody. Scale bars: 3 μm.

Initially, we examined whether target binding and internalization were preserved in ATPPs. As shown in Figure 2A, similar binding of the unconjugated αCDCP1 Ab and the CDCP1-targeting ATPP carrying the EBV_1 peptide (αCDCP1-EBV_1) was detected on CDCP1-expressing MDA-MB231 cells. Moreover, binding of the original Ab and peptide-conjugate resulted in equally efficient target internalization, reaching 50% after 2 h and ~90% after 24 h (Figure 2B). Once internalized, ATPPs should release the conjugated peptides upon disulfide reduction of the SPDP linker in the endosomal compartment. In order to monitor the spatiotemporal release, we generated a disulfide-linked αCDCP1 FRET conjugate. In the non-cleaved condition, excitation of the donor chromophore (BODIPY) at 488 nm results in FRET to the acceptor chromophore (Rhodamine), thereby emitting a red signal. Upon reduction of the disulfide bond, Rhodamine is released from the conjugate, resulting in a green signal (Figure 2C). Utilizing this construct, we showed that the disulfide bond becomes cleaved in endosomes (green signal) but not at the cell surface, where only red FRET signals were visible (Figure 2D). Upon increased incubation time, endosomes steadily enlarged and green fluorescence increased, reflecting accumulation of cleaved constructs. Importantly, separate excitation of the donor and the acceptor chromophores at respectively, 488 and 561 nm (Figure 2E) revealed that the released Rhodamine resides in the endosomal lumen while BODIPY localizes to the cell membrane (Figure 2F), indicating that separation of donor chromophore from the antibody results from disulfide reduction instead of proteolytic digestion. This data is further supported by the finding that BODIPY remained associated with intact αCDCP1 antibodies, as revealed by IgG co-localization (Figure 2G).

### Efficient Presentation of ATPP-Delivered Peptides to CD8+ T-Cells

**Figure 3:**
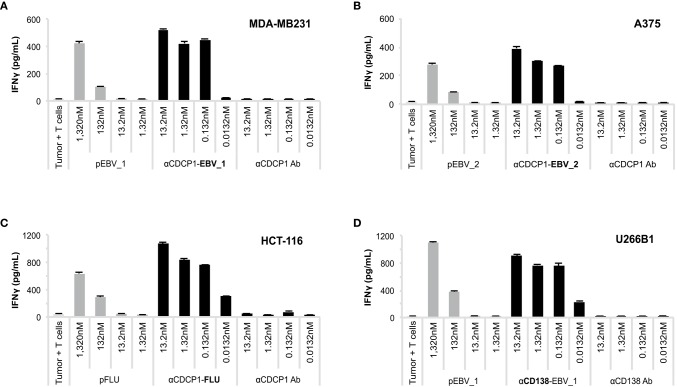
ATPP loaded tumor cells activate peptide-specific CD8+ T-cells. **(A)** Activation of peptide-specific CD8+ T-cells as measured by IFNγ ELISA after treating indicated CDCP1+, HLA-matched cancer cell lines with αCDCP1-EBV_1, **(B)** αCDCP1-EBV_2, or **(C)** αCDCP1-FLU ATPP. Tumor cells exogenously loaded with free peptides (pEBV_1, pEBV_2, pFLU) serve as positive control and unconjugated αCDCP1 Ab as negative control. T-cells were added after 24 h and culture supernatant was harvested after additional 24 h of incubation. **(D)** In a similar manner, CD138-expresssing U266B1 cells were treated with αCD138-EBV_1 ATPP. For each chart, data represent triplicate values and error bars indicate standard deviation. Experiments have been reproduced with 6 different cancer cell lines using 5 different T-cell donors. For additional data please refer to Supplementary Figure 2.

We subsequently investigated whether ATPP-derived peptides released in endosomes are loaded onto MHC class I molecules for recognition by antigen-specific CD8+ T-cells. CDCP1-expressing cancer cells expressing the relevant HLA class I antigens (Supplementary Table 2) were incubated with different concentrations of CDCP1-targeting ATPPs carrying the HLA-A01:01 binding pFLU or the HLA-A02:01 binding pEBV_1 or pEBV_2 epitopes. After 24 h, to allow uptake of the ATPP and loading of peptide antigen into MHC class I, the tumor cells were co-cultured with *in vitro* expanded T-cells highly enriched (>80%) for T-cells specific for the peptides of interest (Supplementary Figure 1). T-cell activation was assessed after another 24 h of co-culture through measurement of IFNγ concentrations in the culture supernatant by ELISA. The three different αCDCP1 ATPPs tested, each loaded with a different peptide epitope, effectively sensitized three different tumor cell lines for recognition by the antigen-specific T-cells at ATPP concentrations as low as 0.132 nM, corresponding to 0.02 μg/mL (Figures 3A–C). The native αCDCP1 Ab did not exhibit any effect at the utilized concentrations. The tumor cells could also be sensitized for T-cell recognition through exogenous loading with the synthetic peptide epitope. Notably, efficient sensitization in this case required 1,320 nM of peptide antigen, 10,000-fold higher concentrations as needed for the corresponding ATPPs. Taking into account the average labeling rate of 1.38 peptides per Ab for the αCDCP1-EBV_1 ATPP (Supplementary Table 1), this corresponds to a >7,200-fold difference in the amount of peptide required to trigger a comparable T-cell response by free peptide vs. ATPP. Importantly, these results could be reproduced for three peptide epitopes in cancer cell lines from various tumor types (Supplementary Figures 2A–G). Moreover, ATPP-mediated sensitization of tumor cells expressing the cell surface proteoglycan CD138 (Syndecan-1) showed similar efficacy (Figure 3D), highlighting the transferability of the ATPP approach to other cancer targets.

### ATPP-Mediated Peptide Loading Into MHC Class I Is Independent of Intracellular Antigen Processing

**Figure 4:**
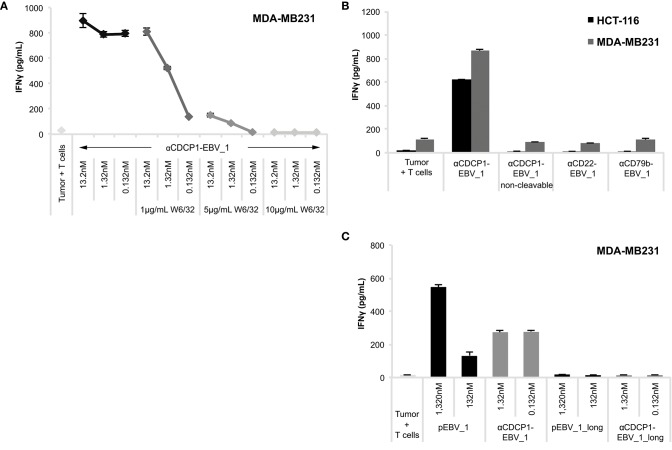
ATPP-mediated peptide delivery is Ab target-dependent and bypasses the antigen processing machinery. Activation of peptide-specific CD8+ T-cells was measured by IFNγ ELISA after co-incubation with CDCP1+, HLA-matched cancer cells that were pre-treated with αCDCP1-EBV_1 ATPP, other ATPPs or synthetic peptide as indicated. **(A)** Accessibility of MHC class I was blocked by addition of pan-HLA class I binding antibody W6/32 at indicated concentrations. **(B)** Sensitization for T-cell recognition by αCDCP1-EBV_1 ATPP was compared with that by an ATPP in which the peptide was bound through a non-cleavable thioether linker, as well as by ATPPs targeting the CD22 or CD79b antigens, which are not expressed on the tumor cells. All compounds were used at 0.132 nM. **(C)** Comparison of T-cell recognition of target cells that were pre-treated with αCDCP1-EBV_1 ATPP comprising the mature (minimal) T-cell epitope, an ATPP comprising an N-terminally extended version of this epitope (EBV_1_long), or the synthetic equivalents of these peptides at concentrations indicated. For each chart, data represent triplicate values and error bars indicate standard deviation. MHC dependency has been shown in 6 (2 donors, 2 cell lines), target dependency in 7 (3 donors, 2 cell lines) and independency of Ag processing in 2 different experiments (2 donors, 2 cell lines). For additional data please refer to Supplementary Figures 2H, 3.

In order to ensure that antigen presentation as induced by ATPPs was MHC class I-restricted and involved the intended pathways as depicted in Figure 1A, we performed a number of control experiments. First, we showed that MHC-blocking with the pan-HLA class I antibody W6/32 abolished ATPP-mediated antigen presentation to T-cells in a concentration-dependent manner (Figure 4A, Supplementary Figure 2H). In further experiments, we demonstrated that disulfide-dependent peptide release from the ATPP is critical for delivery of the peptide into MHC class I. This involved comparison of antigen delivery by ATPPs in which the peptide was linked through the cleavable SPDP-bond vs. a non-cleavable thioether bond. As shown in Figure 4B, the ATPP with the non-cleavable linker failed to sensitize the tumor cells for recognition by the peptide-specific T-cells. Additional controls included in this experiment showed that antigen delivery by cleavable ATPPs strictly depends on surface expression of the Ab-target antigen, in that ATPPs targeting CD22 or CD79b antigens, which are not expressed on the tumor cells used, failed to sensitize these cells for T-cell recognition (Figure 4B). Critical steps in the natural processing of peptide antigens into MHC class I are (i) proteasome cleavage of polypeptide precursors into fragments that result in the generation of peptide epitopes with the correct C-terminus, (ii) followed by transport of these peptide precursors into the endoplasmic reticulum (ER) by the transporter of antigen processing (TAP) complex and (iii) further N-terminal trimming of the precursor by ER-associated aminopeptidases to render a peptide with the optimal length for binding into the MHC class I pocket (2). In order to examine whether our ATPP-delivered peptides could be routed into the classical MHC class I antigen processing pathway, we generated a 15-mer variant of the EBV_1 peptide that was N-terminally extended with the natural 6 amino acids of the LMP2 protein. As shown in Figure 4C (see also Supplementary Figure 3A), the ATPP harboring this elongated peptide (EBV_1_long) failed to sensitize the tumor cells for T-cell recognition, indicating that the endosomal delivery route, as dictated by the cleavable SPDP-linkage, is indeed the main pathway for peptide-loading into MHC class I. This implies that the ATPP-delivered peptide epitopes are directly loaded onto recycling MHC class I molecules.

### Antigen-Specific CD8+ T-Cells Efficiently Kill ATPP-Treated Tumor Cells and Suppress Tumor Growth *in vivo*

**Figure 5:**
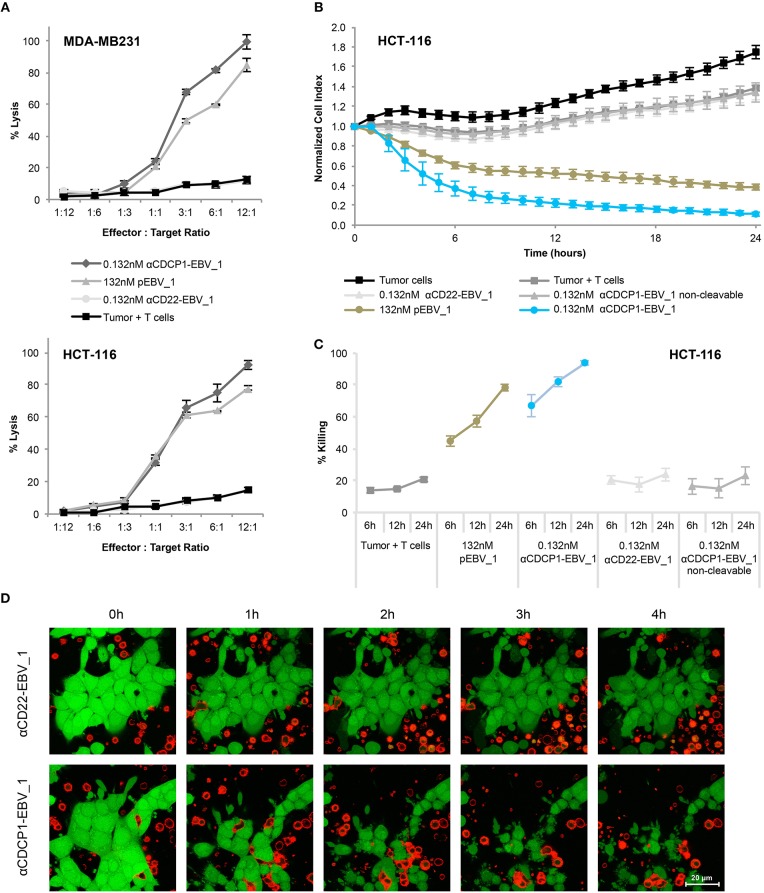
Selective T-cell mediated killing of ATPP-treated tumor cells *in vitro*. **(A)** Killing of CDCP1+, HLA-A02:01+ MDA-MB231 cells by pEBV_1 specific CD8+ T-cells at varying effector-to-target ratios after incubation with αCDCP1-EBV_1, non-targeting αCD22-EBV_1 ATPP or free peptide (pEBV_1). Percentage of lysis was determined by LDH quantification in the supernatant after 24 h. **(B)** Real-time analysis of target cell killing in the xCELLigence system using CDCP1+, HLA-A02:01+ HCT-116 cells, which were pre-incubated for 24 h with indicated ATPPs or synthetic peptides. pEBV_1-specific CD8+ T-cells were added at t = 0 at an effector-to-target ratio of 3:1. **(C)** Percentage of tumor cell killing was calculated with data received from **(B)** at indicated time-points. **(D)** Time-lapse microscopy of co-cultures of CMFDA-labeled HCT-116 cells (green) and PKH-26-labeled pEBV_1-specific CD8+ T-cells (red). HCT-116 were pre-treated with 0.132 nM αCDCP1-EBV_1 ATPP or control αCD22-EBV_1 ATPP. T-cells were added at an effector-to-target ratio of 2:1. For each chart, data represent triplicate values and error bars indicate standard deviation. ATPP mediated killing of target cells vs. non-targeting and non-cleavable constructs has been shown in 4 (2 donors, 2 cell lines) for LDH release and in 13 different experiments (3 donors, 2 cell lines) employing the xCELLigence system.

**Figure 6:**
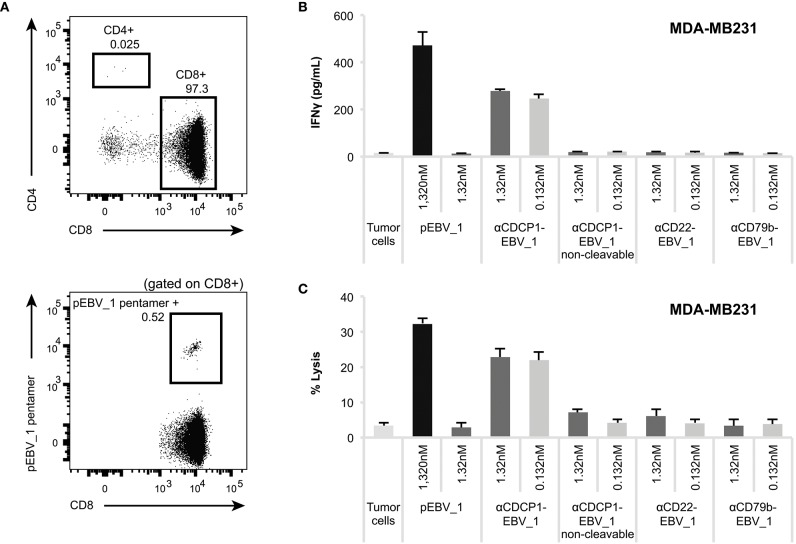
ATPPs can mediate IFNγ secretion and killing by freshly isolated memory CD8+ T-cells. **(A)** Flow cytometric analysis of freshly isolated CD8+ T-cells for the frequency of pEBV_1-specific T-cells by means of pentamer staining. **(B)** Activation of freshly isolated CD8+ T-cells as measured by IFNγ ELISA by αCDCP1-EBV_1 ATPP-loaded MDA-MB231 tumor cells. Free peptide (pEBV_1) serves as reference and non-cleavable αCDCP1- as well as non-targeting αCD22- and αCD79b-ATPP as controls. **(C)** Killing of MDA-MB231 tumor cells by freshly isolated CD8+ T-cells after pre-treatment with indicated ATPPs or synthetic peptides. Total CD8+ T-cell-to-target ratio was 20:1, meaning that pEBV_1-specific CD8+ T-cell-to-target ratio was ~1:10. For each chart, data represent triplicate values and error bars indicate standard deviation.

Having demonstrated that ATPP-treated tumor cells selectively activate peptide-specific CD8+ T-cells, we examined whether this activation resulted in tumor cell killing. Indeed, cell lines treated with αCDCP1-EBV_1 ATPP were efficiently lysed by antigen-specific CD8+ T-cells (Figure 5A). These findings were corroborated by experiments using the xCELLigence system, which allows continuous monitoring of target cell lysis via impedance-based measurement of adherent tumor cells (Figures 5B,C). In accordance with the T-cell sensitization assays (Figure 4), ATPP-mediated peptide loading was superior to pulsing of the target cells with synthetic peptide, while tumor cells treated with αCD22-EBV_1 ATPP or the αCDCP1-EBV_1 ATPP with the non-cleavable linker were not killed. Furthermore, ATPPs carrying the N-terminally extended pEBV_1_long peptide also did not confer target cell lysis (Supplementary Figures 3B,C). The efficiency and selectivity of ATPP treatment was further illustrated in a time-lapse live-cell microscopy experiment comparing αCDCP1-EBV_1 and αCD22-EBV_1 ATPPs (Figure 5D). While these data demonstrate that ATPP treatment can effectively sensitize tumor cells for killing by *in vitro* expanded effector T-cell populations comprising up to 90% peptide-specific T-cells (Supplementary Figure 1), we further evaluated whether ATPP-mediated antigen presentation could trigger the activation of more physiologically relevant frequencies of antigen-specific memory T-cells. For this purpose, we repeated our experiments with CD8+ T-cell populations that were freshly isolated from healthy donor PBMCs, of which only a small fraction (~0.5%) was specific against pEBV_1 (Figure 6A). In spite of this low frequency, recognition of tumor cells treated with αCDCP1-EBV_1 ATPP could readily be detected by IFNγ ELISA (Figure 6B). Interestingly, at this low effector-to-target ratio of ~1:10, we could even detect a significant >20% lysis of the cells treated with αCDCP1-EBV_1 ATPP (Figure 6C), further substantiating the high efficiency by which ATPPs can load tumor cells with specific T-cell epitopes.

**Figure 7:**
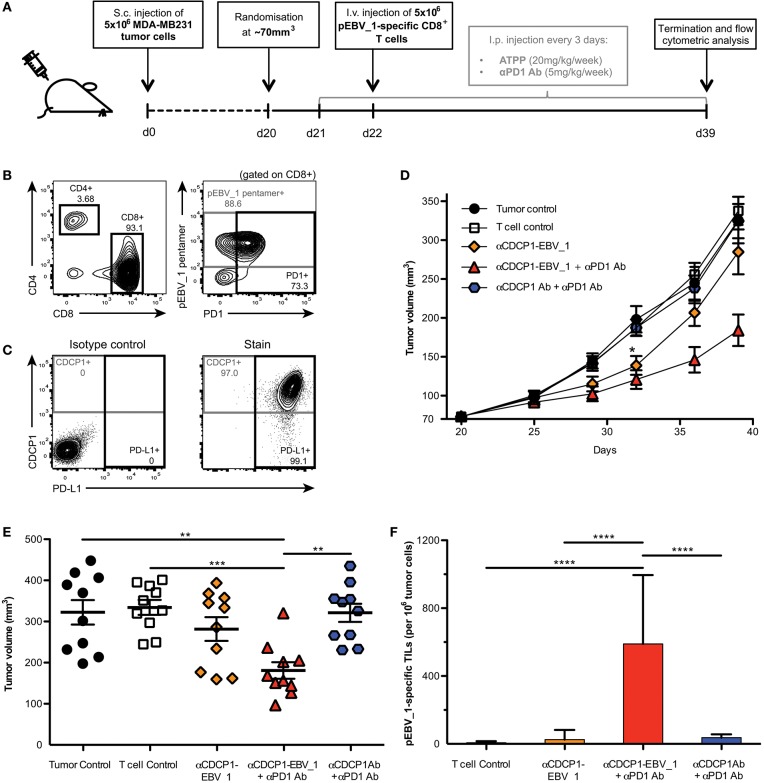
ATPPs efficiently recruit peptide-specific T-cells into the tumor and mediate suppression of tumor growth *in vivo*. **(A)** Study outline, using the CDCP1+, HLA-A02:01+ MDA-MB231 s.c. breast cancer xenograft model in NOG mice and adoptive transfer of *in vitro-*expanded pEBV_1-specific human CD8+ T-cells. **(B)** Flow cytometric phenotyping of T-cells prior to transfer regarding peptide specificity, CD4, CD8, and PD1 expression. **(C)** Analysis of target (CDCP1) and PD-L1 expression in s.c. MDA-MB231 tumors by flow cytometry. **(D)** Kinetics of MDA-MB231 tumor growth as determined by caliper measurement. Mice were either only injected s.c. with tumor cells (Tumor control), additionally received i.v. T-cells (T-cell control) and/or were treated with 20 mg/kg/week αCDCP1-EBV_1 ATPP, αCDCP1 antibody (Ab), and/or 5 mg/kg/week αPD1 Ab. **(E)** Endpoint analysis of tumor volume on day 39. **(F)** Analysis of pEBV_1-specific CD8+ tumor infiltrating lymphocytes (TILs) in s.c. MDA-MB231 tumors after study termination. Data was acquired by means of flow cytometry using peptide-MHC pentamers. For each chart, data is shown as mean and error bars indicate standard error of mean (*n* = 10). The *p*-values represent comparisons between groups using one-way ANOVA followed by Tukey's multiple comparison test. ^**^p < 0.01, ^***^p < 0.001, ^****^p < 0.0001.

In order to examine the impact of ATPP-mediated antigen-loading *in vivo*, we performed a tumor xenograft study in NOG mice (Figure 7A). Mice with established CDCP1+ MDA-MB231 tumors, were infused with *in vitro* expanded, pEBV_1-specific human CD8+ T-cells. Since these T-cells were mostly PD1+ (Figure 7B), while the tumor cells expressed high levels of PD-L1 (Figure 7C), we included an αPD1 Ab in the treatment. Last but not least, we dosed the mice with αCDCP1-EBV_1 ATPP. As shown in Figures 7D,E, this treatment resulted in a statistically significant tumor growth inhibition (>60%) after 20 days of treatment. *In vivo* blockade of PD-1 was important, in that treatment with ATPP alone, although having an initial impact, did not result in sustained suppression of tumor growth. Animals receiving the native αCDCP1 control Ab in combination with αPD1 treatment displayed similar tumor growth kinetics as non-treated animals, showing that treatment with αCDCP1-EBV_1 ATPP was essential to sensitize the tumors for T-cell attack. Flow cytometric analysis of the tumors at the end of the experiment showed that inhibition of tumor growth by the αCDCP1-EBV_1 ATPP/αPD1 combination treatment was associated with strong infiltration of the tumor with pEBV_1-specific T-cells, and that this infiltration was not observed in animals treated with each of the single drugs (Figure 7F). Furthermore, we verified that ATPP-treatment did not result in an overall increase in the MHC Class I surface expression levels (data not shown), indicating that the ATPP-mediated delivery of the peptide epitope was instrumental in the sensitization for T-cell recognition. Importantly, no weight loss or any other signs of toxicity were observed in any of the treated groups.

## Discussion

In addition to the immunosuppressive tumor microenvironment, which can be counteracted by checkpoint inhibitors and other immunomodulators (20, 21), the lack of highly immunogenic antigens represents a major hurdle in the development of effective T-cell therapy for solid cancers (22). The present study describes a new class of T-cell epitope conjugated antibodies, called ATPPs, that provide a flexible platform for the efficient delivery of immunogenic T-cell epitopes into the MHC class I of tumors. In this manner, tumors can be sensitized for the attack by pre-existing, virus-specific memory T-cells.

The design of ATPPs allows for the delivery of various MHC-I-restricted peptides from common pathogens with one pre-requisite: the presence of a cysteine residue in the T-cell epitope sequence, which is required for disulfide-dependent conjugation to the antibody. Our data show that any mature MHC-I peptide containing a cysteine, independent of its position in the peptide sequence, can be conjugated. The flexibility is advantageous for the clinical applicability of the ATPP technology, because it enables epitope delivery in tumors of patients with diverse HLA-types. Preferentially selected peptide epitopes bind to highly prevalent HLA allotypes like HLA-A01:01 or -A02:01 (23), and are derived from viruses with high prevalence in humans such as EBV (24) or CMV (25). Furthermore, the design of ATPPs allows for the conjugation of multiple peptides per antibody while only marginally increasing the molecular size. This offers the option of loading antibodies with a higher dose and/or a mixture of different peptide epitopes. The use of ATPPs armed with multiple peptides is likely to increase the efficacy of the T-cell attack and diminish the likelihood of tumor immune escape through the loss of selected MHC class I molecules (26). Notably, the MHC class I antigen presentation in tumors is often compromised due to suppression of the antigen processing machinery (27). The ATPP design offers a further advantage in this respect, in that it renders peptide loading into MHC Class I independent of this pathway. While the efficiency of ATPP-mediated epitope delivery to tumor cells *in vitro* is close to 100%, it is conceivable that *in vivo* treatment of tumor-bearing mice results in epitope delivery to only a fraction of the tumor cells. As such, the complete T-cell mediated clearance of tumors will be dependent of spreading of the T-cell response to endogenously expressed antigens (28).

Although known tumor antigens, including neoantigens and tumor-associated auto-antigens, could also be delivered by ATPPs, the utilization of pathogen-derived peptides, especially common viral epitopes, has several advantages. First, the T-cell repertoire against viral antigens is not blunted by thymic tolerance and/or chronic antigen exposure in the tumor microenvironment. Instead, ATPPs loaded with viral epitopes mediate recruitment of high affinity memory T-cells that are able to launch a robust immune effector response capable of surmounting the immunosuppressive mechanisms in the tumor microenvironment. Secondly, directing the immune response against non-self antigens reduces the risk for autoimmune pathology due to on-target side-effects. Thirdly, the potential of viral antigens in mediating tumor rejection is well-documented (29) and T-cell mediated targeting of cancer through viral epitopes was already proven to be successful (30–33). Last but not least, two independent studies recently provided evidence that the natural T-cell infiltrate of tumors with non-viral etiology contains significant numbers of pathogen-specific T-cells, including EBV-, CMV- and Influenza-specific T cells (34, 35), which are apparently activated by pathogen challenge elsewhere in the body and migrate into the chronically inflamed tumor tissue. While in absence of their cognate antigen, these T-cells will neither exert effector activity, nor become exhausted, they would readily engage in an effector response in ATPP-treated tumors.

Several alternative strategies have been reported toward increasing tumor immunogenicity by delivering viral antigens through T-cell epitope-loaded antibodies (36–38). Kang et al. developed a tumor-targeting antibody conjugate in which the immunogenic CD8+ T-cell epitope is linked through a furin cleavage site (36). As a result, the peptide epitope is released into the tumor microenvironment where it can bind to MHC class I through exogenous loading. On the one hand, a potential advantage of this strategy is that this may also sensitize other cells in the tumor microenvironment to T-cell attack. On the other hand, the process of exogenous loading of T-cell epitopes into MHC is relatively inefficient, as demonstrated by the experimental data shown in our present study. The antigen-armed antibodies developed by Delecluse and coworkers are more similar to our ATPPs, in that these target tumor surface antigens that are internalized upon antibody binding, resulting in release of the T-cell epitope in the endosomal pathway (37, 38). The key differences with our approach are that the T-cell epitope concerned is MHC class II restricted and that release of the epitope from the immunoglobulin molecule is dependent on antigen processing by the tumor cell. Overall, this strategy appears more suitable for targeting hematopoietic malignancies, as demonstrated in these publications. A very recent report demonstrates the sensitization of tumors for attack by T-cells through the repeated, intratumoral injection of synthetic, viral peptide epitopes (39). While the *in vivo* results of these studies are very similar to ours, in that peptide delivery sensitizes tumors to immune checkpoint blockade, the feasibly and safety of repeated intratumoral peptide delivery in the clinical setting is a matter of debate. Finally, the exploitation of the pre-existing, antigen-specific memory T-cell repertoire clearly differentiates ATPPs from bispecific T-cell engagers (BiTEs). BiTEs stimulate a broad diversity of CD3-expressing cells, involving the risk for adverse events related to T-cell dependent hypersensitivity reactions and cytokine release syndromes (40). In contrast, ATPPs recruit a well-defined pool of T-cells with pre-defined antigen specificity.

In conclusion, ATPP immunoconjugates constitute a promising new drug format toward overcoming immune tolerance by recruiting highly activated, non-exhausted effector T-cells into the tumor microenvironment. The application of ATPPs in conjunction with immune checkpoint blockade may offer a path toward increasing clinical response rates and sensitizing cancers with lower mutational load for this therapy.

## Data Availability

The raw data supporting the conclusions of this manuscript will be made available by the authors, without undue reservation, to any qualified researcher.

## Ethics Statement

The animal study was reviewed and approved by Regierung von Oberbayern.

## Author Contributions

SD, LH, VL, and ALi: conception and design of ATPP molecules. JS, VL, and ALi: experimental conception and design. JS, LH, OM, GT, ALe, VL, and ALi: development of methodology and analysis and interpretation of data. JS, KM, DZ-L, HS, DK, and IF: acquisition of data. JS and RO: writing of the manuscript. JS, LH, OM, RO, VL, and ALi: review and revision of the manuscript. RO, VL, and ALi: study supervision.

### Conflict of Interest Statement

During the study, all authors except for RO were employed by F. Hoffman-La Roche AG and received salary. Other than study funding, employee salaries, and approval of the final manuscript, the funding organization had no influence on: the study design, data collection, data analysis, data interpretation, or preparation of the manuscript. SD, LH, VL, and ALi have ownership interest in an issued patent (WO2016131856). The remaining authors declare that the research was conducted in the absence of any commercial or financial relationships that could be construed as a potential conflict of interest.
