# Supplementary material for: Sensitization of Tumors for Attack by Virus-Specific CD8+ T-Cells Through Antibody-Mediated Delivery of Immunogenic T-Cell Epitopes
Source: Front Immunol. 2019 Aug 21;10:1962. doi: 10.3389/fimmu.2019.01962 (PMC6712545; doi:10.3389/fimmu.2019.01962)
Supplement: Supplementary file 1 [file Table_1.DOCX]

**Supplementary Table 1 – Peptide labeling rates of ATPP conjugates**

| **ATPP conjugate** | **Average peptide per antibody** |
| --- | --- |
| αCDCP1-EBV_1 | 1.38 |
| αCDCP1-EBV_1 non cleavable | 1.95 |
| αCDCP1-EBV_1_long | 0.60 |
| αCDCP1-EBV_2 | 1.83 |
| αCDCP1-FLU | 5.35 |
| αCD22-EBV_1 | 2.44 |
| αCD79b-EBV_1 | 2.81 |
| αCD138-EBV_1 | 1.66 |

Peptide labeling rates were determined by mass spectrometric analysis using

ESI-TOF-MS measurement and detection.
