# Supplementary material for: Sensitization of Tumors for Attack by Virus-Specific CD8+ T-Cells Through Antibody-Mediated Delivery of Immunogenic T-Cell Epitopes
Source: Front Immunol. 2019 Aug 21;10:1962. doi: 10.3389/fimmu.2019.01962 (PMC6712545; doi:10.3389/fimmu.2019.01962)
Supplement: Supplementary file 2 [file Table_2.DOCX]

**Supplementary Table 2 – CDCP1 and HLA expression of cell lines**

| **Cell line** | **CDCP1** | **HLA-A2** | **HLA-A1/A11/A26** | **HLA-A1/A36** |
| --- | --- | --- | --- | --- |
| MDA-MB-231 | 36,366 | 147,187 *(A02*01, A02*17)* | - | - |
| HCT-116 | 47,678 | 7,990 *(A02*01)* | 24,679 *(A01*01)* | 1,747 *(A01*01)* |
| A-375 | 1,736 | 8,629 *(A02*01)* | 21,758 *(A01*01)* | 710 *(A01*01)* |
| PC-3 | 43,488 | - | 24,452 *(A01*01)* | 1,336 *(A01*01)* |
| BxPC-3 | 16,632 | - | 35,430 *(A01*01)* | 2,069 *(A01*01)* |

Values represent mean fluorescence intensity (MFI) minus MFI of the isotype control, as acquired by flow cytometry. Brackets indicate relevant HLA genotypes.
