# Supplementary material for: Sensitization of Tumors for Attack by Virus-Specific CD8+ T-Cells Through Antibody-Mediated Delivery of Immunogenic T-Cell Epitopes
Source: Front Immunol. 2019 Aug 21;10:1962. doi: 10.3389/fimmu.2019.01962 (PMC6712545; doi:10.3389/fimmu.2019.01962)
Supplement: Supplementary file 3 [file Data_Sheet_1.PDF]

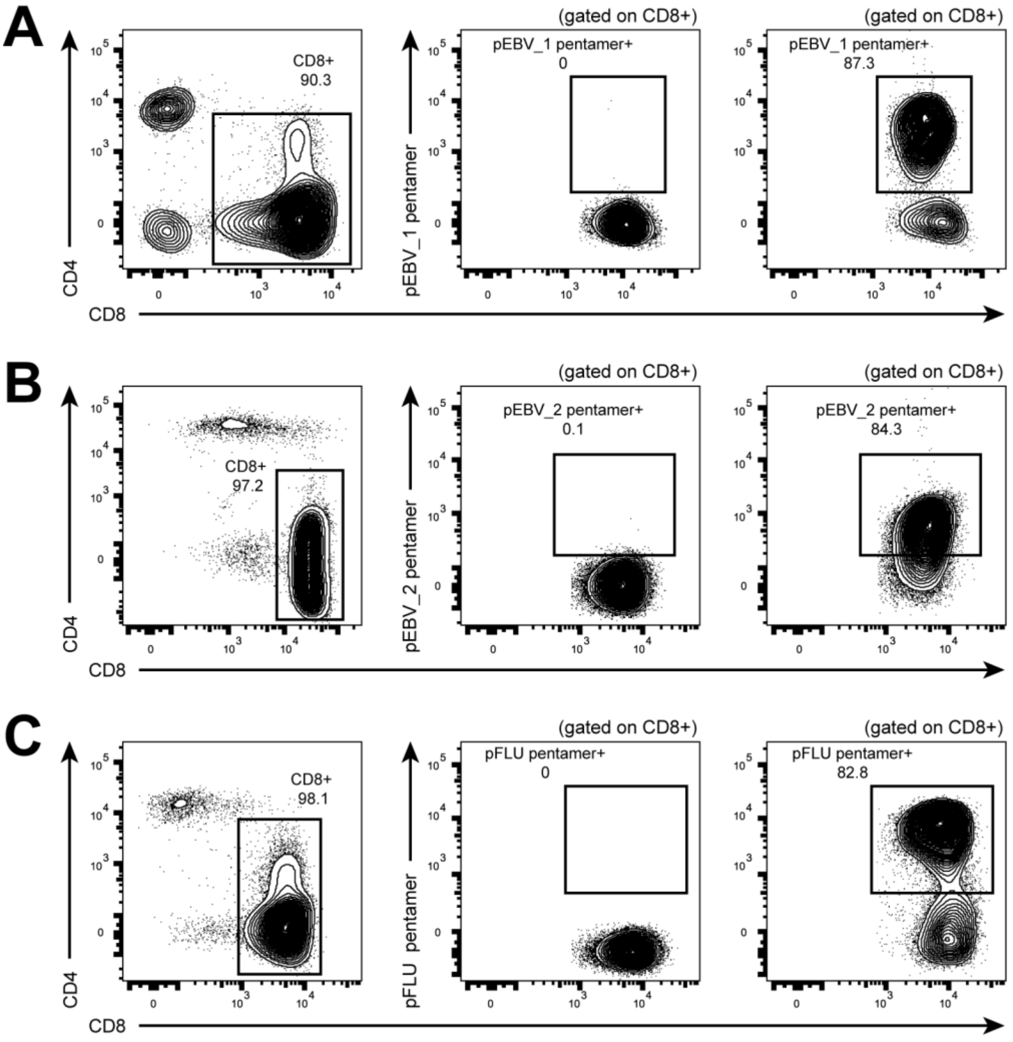

Supplementary Figure 1 – Peptide-specificity of T cells after *in vitro* expansion. Representative flow cytometric analysis of *in vitro* expanded T cells by means of peptide-MHC pentamers after culture with (**A**) pEBV_1, (**B**) pEBV_2 or (**C**) pFLU. Cells were gated on single, live, CD3+ and CD8+ (CD4-, left panel). Fluorescence minus one (middle) versus pentamer stained cells (right) are shown.
