# Supplementary material for: Sensitization of Tumors for Attack by Virus-Specific CD8+ T-Cells Through Antibody-Mediated Delivery of Immunogenic T-Cell Epitopes
Source: Front Immunol. 2019 Aug 21;10:1962. doi: 10.3389/fimmu.2019.01962 (PMC6712545; doi:10.3389/fimmu.2019.01962)
Supplement: Supplementary file 4 [file Data_Sheet_2.PDF]

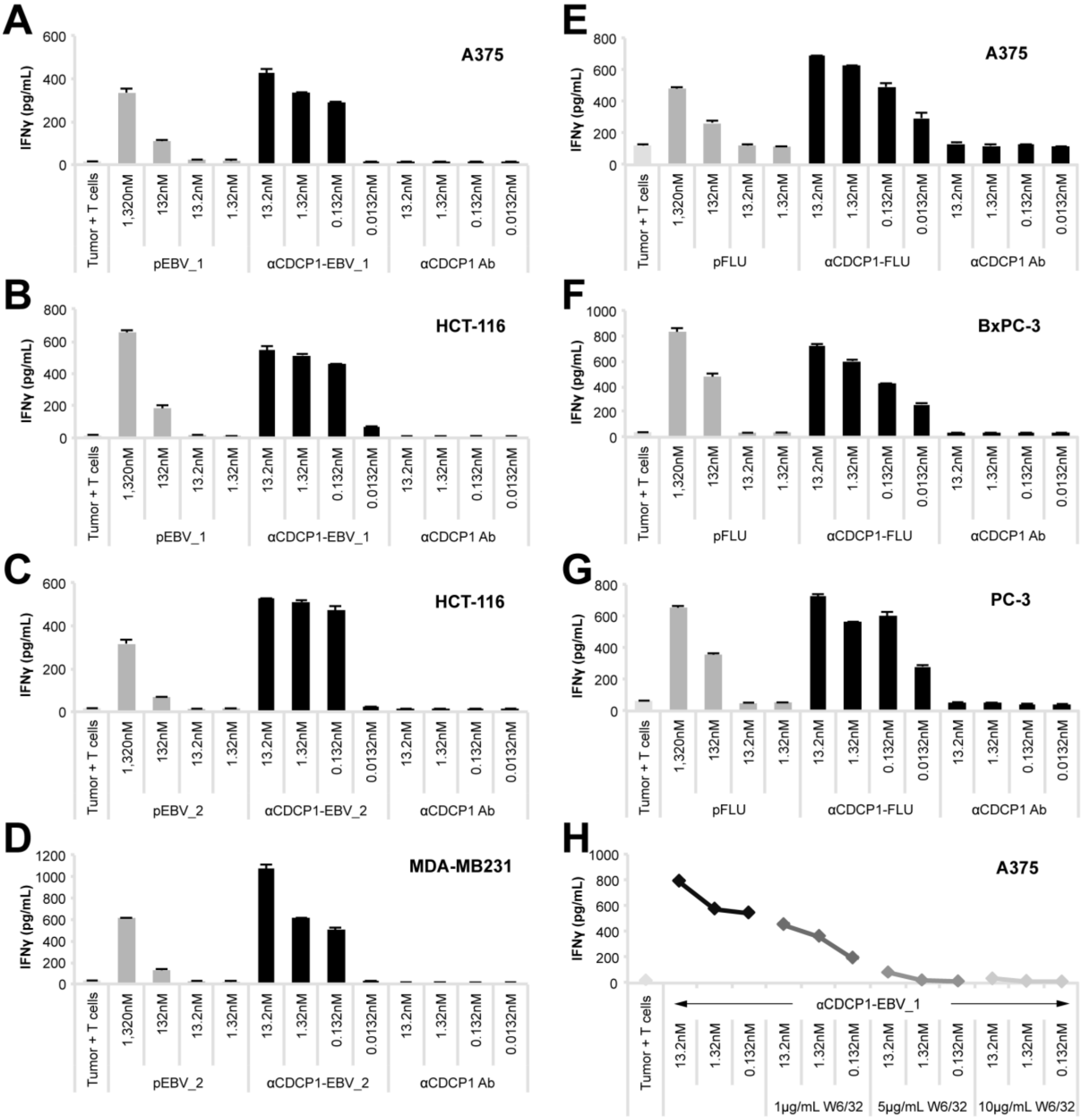

Supplementary Figure 2 – Demonstration of ATPP-mediated T-cell activation in different tumor types.

Activation of *in vitro*-expanded peptide-specific CD8+ T-cells as measured by IFNγ ELISA after treatment of indicated CDCP1+, HLA-matched cancer cell lines with (**A, B**) αCDCP1-EBV_1, (**C, D**) αCDCP1-EBV_2 or (**E-G**) αCDCP1-FLU ATPP. Free peptides (pEBV_1, pEBV_2, pFLU) serve as reference and unconjugated αCDCP1 Ab as control. (**H**) Blocking of the MHC accessibility by means the MHC-I binding antibody clone W6/32. For each chart, data represent triplicate values and error bars indicate standard deviation.
