# Supplementary material for: Sensitization of Tumors for Attack by Virus-Specific CD8+ T-Cells Through Antibody-Mediated Delivery of Immunogenic T-Cell Epitopes
Source: Front Immunol. 2019 Aug 21;10:1962. doi: 10.3389/fimmu.2019.01962 (PMC6712545; doi:10.3389/fimmu.2019.01962)
Supplement: Supplementary file 5 [file Data_Sheet_3.PDF]

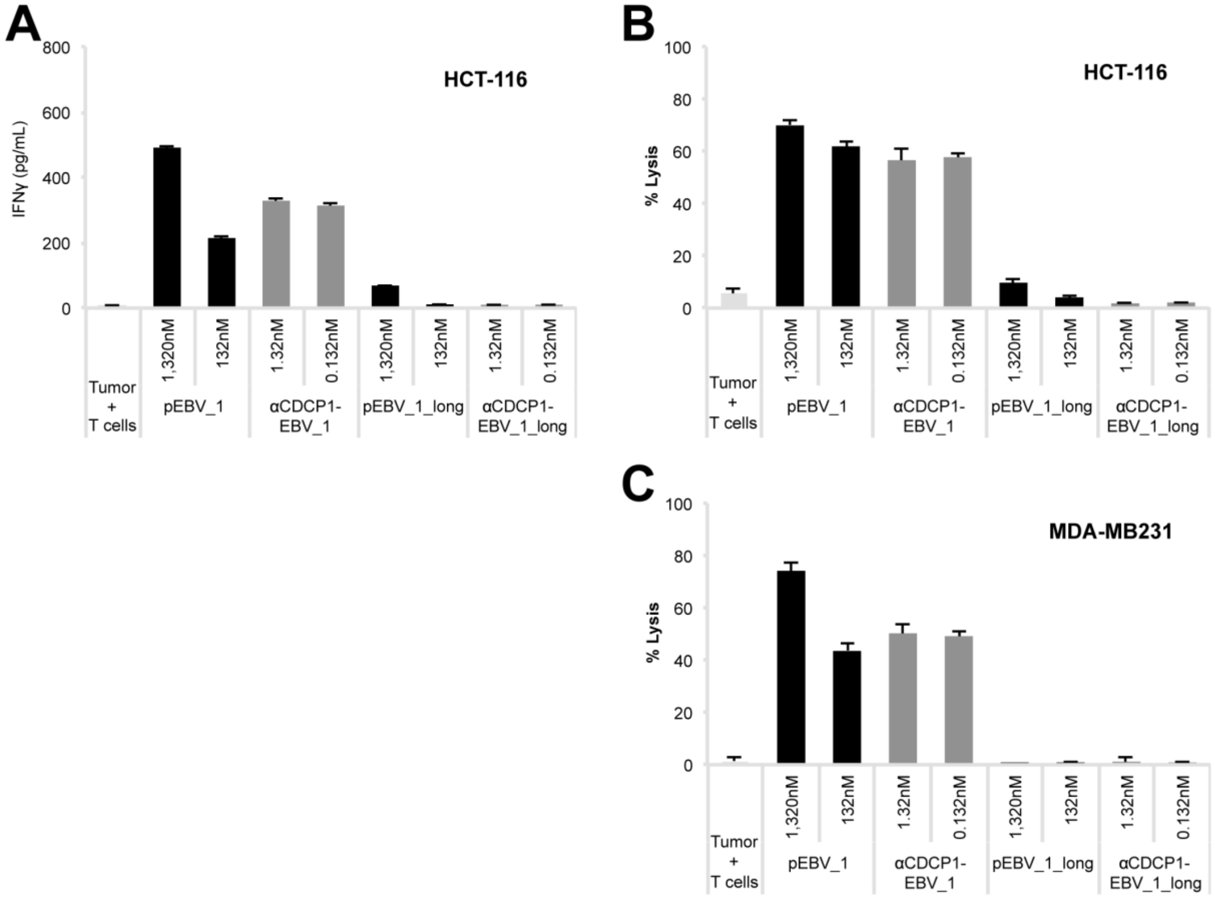

Supplementary Figure 3 – ATPPs carrying N-terminally extended MHC-I peptides do not mediate efficient epitope presentation.

(**A**) Activation of *in vitro*-expanded peptide-specific CD8+ T-cells as measured by IFNγ ELISA or (**B, C**) lysis of target cells after treatment of indicated CDCP1+, HLA-matched cancer cells with αCDCP1 ATPP comprising either the mature (minimal) pEBV_1 or N-terminally extended pEBV_1_long peptide. Percentage of lysis was determined by LDH quantification in the supernatant after 24 hours. Effector-to-target ratio 3:1. For each chart, data represent triplicate values and error bars indicate standard deviation.
